# The adaptor protein Disabled-2: new insights into platelet biology and integrin signaling

**DOI:** 10.1186/s12959-016-0101-5

**Published:** 2016-10-04

**Authors:** Hui-Ju Tsai, Ching-Ping Tseng

**Affiliations:** 1 Department of Medical Biotechnology and Laboratory Science, Collage of Medicine, Chang Gung University, Kweishan, Taoyuan 333, Taiwan, Republic of China; 2 Molecular Medicine Research Center, Chang Gung University, Kweishan, Taoyuan 333, Taiwan, Republic of China; 3 Graduate Institute of Biomedical Science, Collage of Medicine, Chang Gung University, Kweishan, Taoyuan 333, Taiwan, Republic of China; 4 Department of Laboratory Medicine, Chang Gung Memorial Hospital, Kweishan, Taoyuan 333, Taiwan, Republic of China

**Keywords:** Disabled-2, Integrin αIIbβ3, Megakaryocyte, Platelet

## Abstract

Multiple functions of platelets in various physiological and pathological conditions have prompted considerable attention on understanding how platelets are generated and activated. Of the adaptor proteins that are expressed in megakaryocytes and platelets, Disabled-2 (Dab2) has been demonstrated in the past decades as a key regulator of platelet signaling. Dab2 has two alternative splicing isoforms p82 and p59. However, the mode of Dab2’s action remains to be clearly defined. In this review, we highlight the current understanding of Dab2 expression and function in megakaryocytic differentiation, platelet activation and integrin signaling. Accordingly, Dab2 is upregulated when the human K562 cells, human CD34^+^ hematopoietic stem cells, and murine embryonic stem cells were undergone megakaryocytic differentiation. Appropriate level of Dab2 expression is essential for fate determination of mesodermal and megakaryocytic differentiation. Dab2 is also shown to regulate cell-cell and cell-fibrinogen adhesion, integrin αIIbβ3 activation, fibrinogen uptake, and intracellular signaling of the megakaryocytic cells. In human platelets, p82 is the sole Dab2 isoform present in the cytoplasm and α-granules. Dab2 is released from the α-granules and forms two pools of Dab2 on the outer surface of the platelet plasma membrane, one at the sulfatide-bound and the other at integrin αIIbβ3-bound forms. The balance between these two pools of Dab2 controls the extent of clotting reaction, platelet-fibrinogen interactions and outside-in signaling. In murine platelets, p59 is the only Dab2 isoform and is required for platelet aggregation, fibrinogen uptake, RhoA-ROCK activation, adenosine diphosphate release and integrin αIIbβ3 activation stimulated by low concentration of thrombin. As a result, the bleeding time is prolonged and thrombus formation is impaired for the megakaryocyte lineage-restricted Dab2 deficient mouse. Although discrepancies of Dab2 function and isoform expression are noted between human and murine platelets, the studies up-to-date define Dab2 playing a pivotal role in integrin signaling and platelet activation. With the new tools such as CRISPR and TALEN in the generation of genetically modified animals, the progress in gaining new insights into the functions of Dab2 in megakaryocyte and platelet biology is expected to accelerate.

## Background

Platelets are the second most abundant blood cells and are derived from the cytoplasm of megakaryocytes [1]. The crucial role of platelet in haemostasis and thrombosis has prompted extensive attentions on unveiling the underlying mechanisms of platelet activation induced by soluble agonists [2–4]. Platelet activation is mainly mediated by binding of ligands to the membrane receptors such as the immunoglobulin family of glycoproteins for collagen and the G-protein coupled receptors for thrombin, thromboxane A2 (TXA_2_) and adenosine diphosphate (ADP) [5]. Collagen interacts with glycoprotein VI which contains an immunoreceptor tyrosine-based activation motif (ITAM). The ITAM is phosphoryated by two Src kinases (Lyn and Fyn) and recruits the protein tyrosine kinase Syk to the plasma membrane for phosphorylation of downstream substrates at the tyrosine residue that are essential for platelet activation [5]. Other soluble agonists such as thrombin, TXA_2_ and ADP bind to the respective G protein-coupled receptors and cause an increase in intracellular calcium and protein kinase C (PKC) activity, Rho activation, inhibition of adenylyl cyclase and activation of phosphoinositide 3-kinase-Akt through the Gα_q_-, Gα_12/13_-, Gα_i_-, and Gβγ-dependent pathway, respectively [5, 6]. The inside-out signaling induced by different platelet agonists activates integrin αIIbβ3 followed by the binding of fibrinogen to integrin αIIbβ3 and activation of outside-in signaling. These intracellular events ultimately lead to platelet activation, secretion and aggregation [7]. Despite extensive studies, the underlying mechanisms of platelet signaling networks still wait to be fully elucidated.

Adaptor protein is a type of proteins mediating protein-protein and protein-lipid interactions. It has been clearly demonstrated that adaptor proteins are essential for coupling membrane receptors to intracellular signaling pathways and the assembly of signaling scaffolds within the cells. Many adaptor proteins expressed in the platelets are involved in inside-out and outside-in signaling of integrin during platelet activation [8]. Disabled-2 (Dab2) is a newly identified adaptor protein that is known to express in megakaryocytes and platelets from a variety of species [9, 10]. The current knowledge about the roles of Dab2 in megakaryocytic differentiation and platelet signaling is still in the beginning. This review will focus on the expression and functional aspects of Dab2 in megakaryocytic differentiation, platelet activation and integrin signaling.

## Review

### Discovery and the protein properties of Dab2

**Fig. 1:**
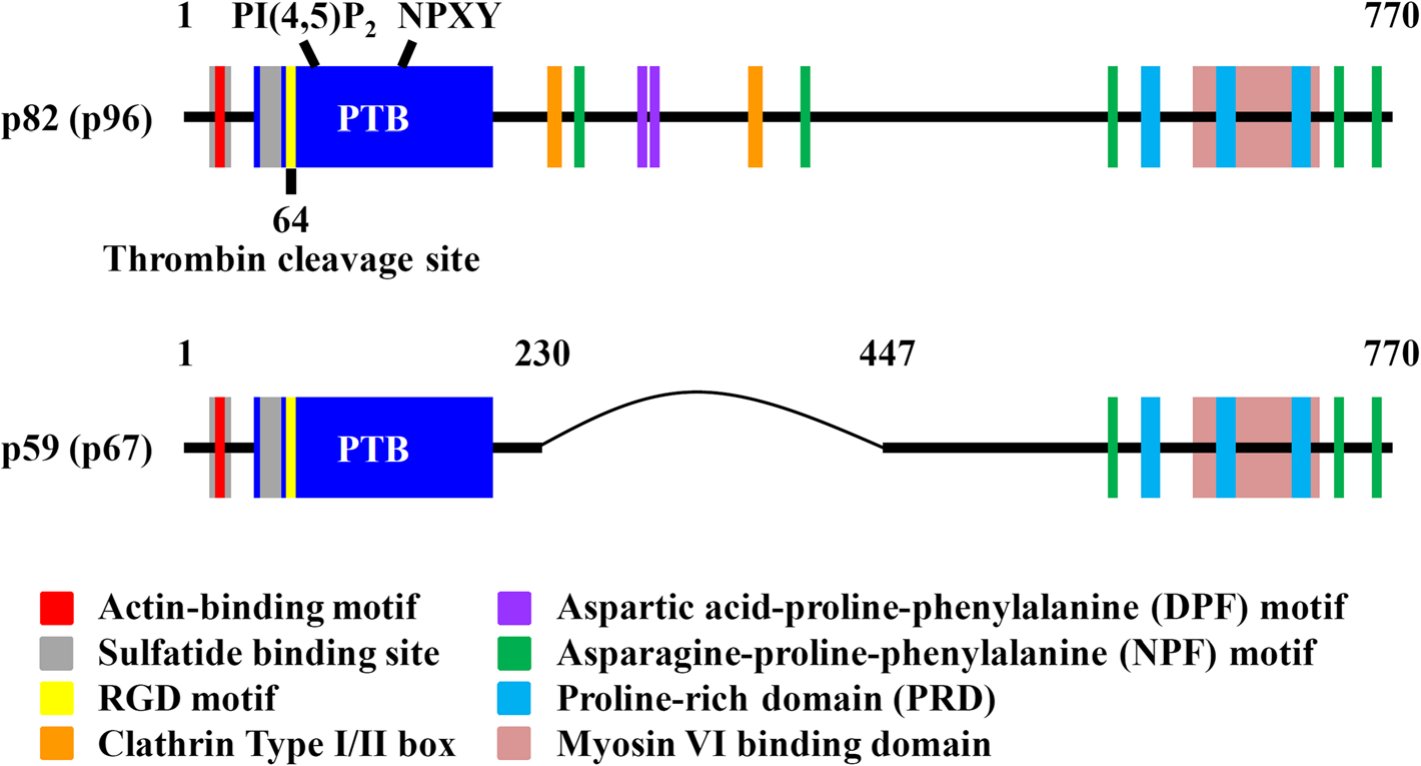
Schematic illustration for the primary protein structure of Dab2. The primary structures for both Dab2 isoforms p82 (p96) and p59 (p67) are shown. The p59 (p67) isoform of Dab2 lacks the ninth coding exon corresponding to the amino acid residues of 230–447 and results in the deletion of several binding sites for endocytic proteins. The N-terminus of Dab2 contains an actin-binding motif (^25^KKEK^28^), two sulfatide binding sites (amino acid residues 24–32 and 49–54), an RGD motif (^64^RGD^66^), one thrombin cleavage site (^64^R) and the PTB domain (amino acid residues 45–196). Dab2-PTB is the binding sites for PI(4,5)P_2_ and the tails of a subset of non-tyrosine-phosphorylated NPXY-containing receptors. The clathrin type I (^236^LVDLN^240^) and type II (^363^PWPFS^367^) box sequences, and the two DPF motifs (^293^DPFRDDPF^300^) are located at the middle region of Dab2 protein. The DPF motifs bind to the α-adaptin subunit of the clathrin adaptor protein AP-2. The five asparagine-proline-phenylalanine (NPF) motifs spanning the middle and C-terminus of Dab2 possibly bind proteins containing Eps homology domain. The C-terminus of Dab2 contains the myosin VI binding domain and the PRD for the binding of proteins containing SH3 domain

Human *dab2* gene is located at the chromosome 5p13 and was first identified by Mok et al. as the tumor suppressor gene of the ovary cancer in 1994 [11]. The mouse Dab2 protein was then revealed in 1995 during the analysis of phosphoproteins induced by colony-stimulating factor-1 (CSF-1) in macrophage [12]. In 1998, Tseng et al. further defined rat *dab2* as the differentially expressed gene that was up-regulated in the castrated rat prostate [13]. At least two Dab2 isoforms with the molecular weight of 82 and 59 kDa, referred to p82-Dab2 and p59-Dab2, respectively, are generated through alternative splicing (Fig. 1) [14]. Because of the undefined post-translational protein modification, the protein bands of p82-Dab2 and p59-Dab2 are up-shifted to the positions at 96 and 67 kDa on sodium dodecyl sulfate-polyacrylamide gel electrophoresis. Hence, p82-Dab2 and p59-Dab2 sometimes are referred to p96-Dab2 and p67-Dab2, respectively. The ninth coding exon corresponding to the amino acids 230–447 of p82-Dab2 is not present in the protein of p59-Dab2. As a result, several binding sites for endocytic proteins are absent in p59-Dab2. Particular motifs mediating protein-protein and protein-lipid interactions are present in Dab2, allowing them to communicate with other signaling molecules. The phosphotyrosine binding (PTB) domain is located at the N-terminus of Dab2, playing a role in the interaction of Dab2 with DIP1/2, Smad2/3, Dishevelled-3, phosphatidylinositol 4,5-bisphosphate (PI(4,5)P_2_), and a subset of receptors such as integrin, low density lipoprotein receptor, megalin and related receptors that contain the non-tyrosine-phosphorylated NPXY motif [14–21]. The aspartic acid-proline-phenylalanine (DPF) motif of Dab2 interacts with the α-adaptin subunit of the clathrin adaptor protein 2 (AP-2) [21]. The C-terminal proline-rich domain (PRD) interacts with Grb2, c-Src, Akt and c-Cbl-interacting protein of 85 kDa [22–26]. By interacting with other cellular factors through these motifs, Dab2 elicits its functions in endocytosis, differentiation, and immune response and is involved in the cell signaling pathways of Ras-mitogen activated protein kinase (MAPK), Wnt, TGF-β, c-Src and RhoA-ROCK [24, 27–36]. Dab2 is also known to regulate cytoskeleton reorganization by binding to non-muscle myosin heavy chain IIA, myosin VI, actin, and dynein [12, 37–39].

Dab2 is a phosphoprotein with several phosphoryation sites having been identified. Dab2 is phosphorylated at serine residues in murine macrophage cell line in response to mitogenic stimulation by CSF-1 [12]. Dab2-Ser24 is phosphorylated by PKCβII, γ and δ but not by casein kinase II, playing a critical role in the inhibition of 12-O-tetradecanoylphorbol-13-acetate (TPA)-induced AP-1 activity and integrin activation [33, 40]. Dab2 is hyperphosphorylated by the cyclin-dependent serine/threonine kinase Cdc2 during the mitosis phase of the cell cycle in HeLa S3 cells [41]. The phosphorylated Dab2 interacts with the peptidylprolyl isomerase Pin1 that facilitates Dab2 dephosphorylation immediately after the end of mitosis phase [42]. Similarly, Akt interacts with PRD domain of Dab2 and phosphorylates Ser448/Ser449 to regulate albumin endocytosis and mediate albumin uptake in proximal tubule [25, 43]. These distinct protein properties facilitate the involvement of Dab2 in diverse signaling network in response to extracellular responses.

### Expression pattern of Dab2 in megakaryocytes and platelets

The first study addressing Dab2 expression and function in megakaryocytes and platelets was published in 2001 [31]. Dab2 is upregulated when the human leukemic K562 cells, human CD34^+^ hematopoietic pluripotent stem cells, and murine embryonic stem cells (ESCs) are undergone megakaryocytic differentiation (Table 1) [31–33, 35]. Among the platelets from the species of murine, rat, and human, murine platelets have the least amount of Dab2 [9]. This is in accord with the genome-wide RNA-seq analysis of platelet transcriptomes that revealed several thousands-fold differences for the expression of Dab2 transcripts between human and mouse platelets [44]. Moreover, Dab2 isoforms are differentially expressed in human, rat and murine platelets. Both p82-Dab2 and p59-Dab2 are detectable in the rat platelets, while p82-Dab2 and p59-Dab2 is mainly expressed in the platelets from human and murine, respectively [9].

**Table 1:** Dab2 expression and function in megakaryocytes and platelets

Experimental systems	Reported Dab2 expression/function	References
Human K562 cells	Increased Dab2 expression during TPA-induced megakaryocytic differentiation	[19, 31–34]
	Positive regulation of fibrinogen uptake	
	Dab2 interacts with integrin β3 and inhibits integrin αIIbβ3 activation	
Human CD34^+^ stem cells	Increased Dab2 expression during TPO-induced megakaryocytic differentiation	[10]
Mouse embryonic stem cells/OP9 co-culture	Increased Dab2 expression during mesodermal and megakaryocytic differentiation	[35]
	Dab2 is required for mesodermal differentiation	
Human platelets	High expression of p82-Dab2 in the cytoplasm and α-granule	[10, 33, 47–49]
	Dab2 interacts with the cytoplamic tail of platelet integrin	
	Secreted Dab2 interacts with integrin αIIb and sulfatide; is a substrate of thrombin	
	Dab2 regulates fibrinogen binding and homotypic and heterotypic platelet interactions	
Mouse platelets	Low expression of p59-Dab2	[9]
	Dab2-deficient mice display a prolonged bleeding time and impaired thrombus formation	
	Dab2 is required for platelet aggregation, fibrinogen uptake, RhoA-ROCK activation, ADP release and integrin αIIbβ3 activation stimulated by low concentration of thrombin	

The evolutionary roles for an increase in Dab2 expression from mouse to rat and human platelets and the species-specific expression of Dab2 isoforms are not yet understood. Distinctive functions of p82-Dab2 and p59-Dab2 have been unveiled in several studies. p82-Dab2 is known to regulate receptor-mediated endocytosis, while p59-Dab2 is a transcriptional regulator when the F9 cells are undergone differentiation [18, 20, 45, 46]. Knock-in expression of p59-Dab2 only partially compromises the absence of Dab2 in the Dab2-knockout mice [14]. The increased expression of p82-Dab2 protein in human platelets may fine tune platelet response to soluble agonists and provide a superior way to prevent excessive blood loss in the large mammals. Future study using an in vivo animal model expressing human platelet p82-Dab2 should provide new insight for the aforementioned hypothesis.

### Dab2 functions in megakaryocytic differentiation and platelet signaling

**Fig. 2:**
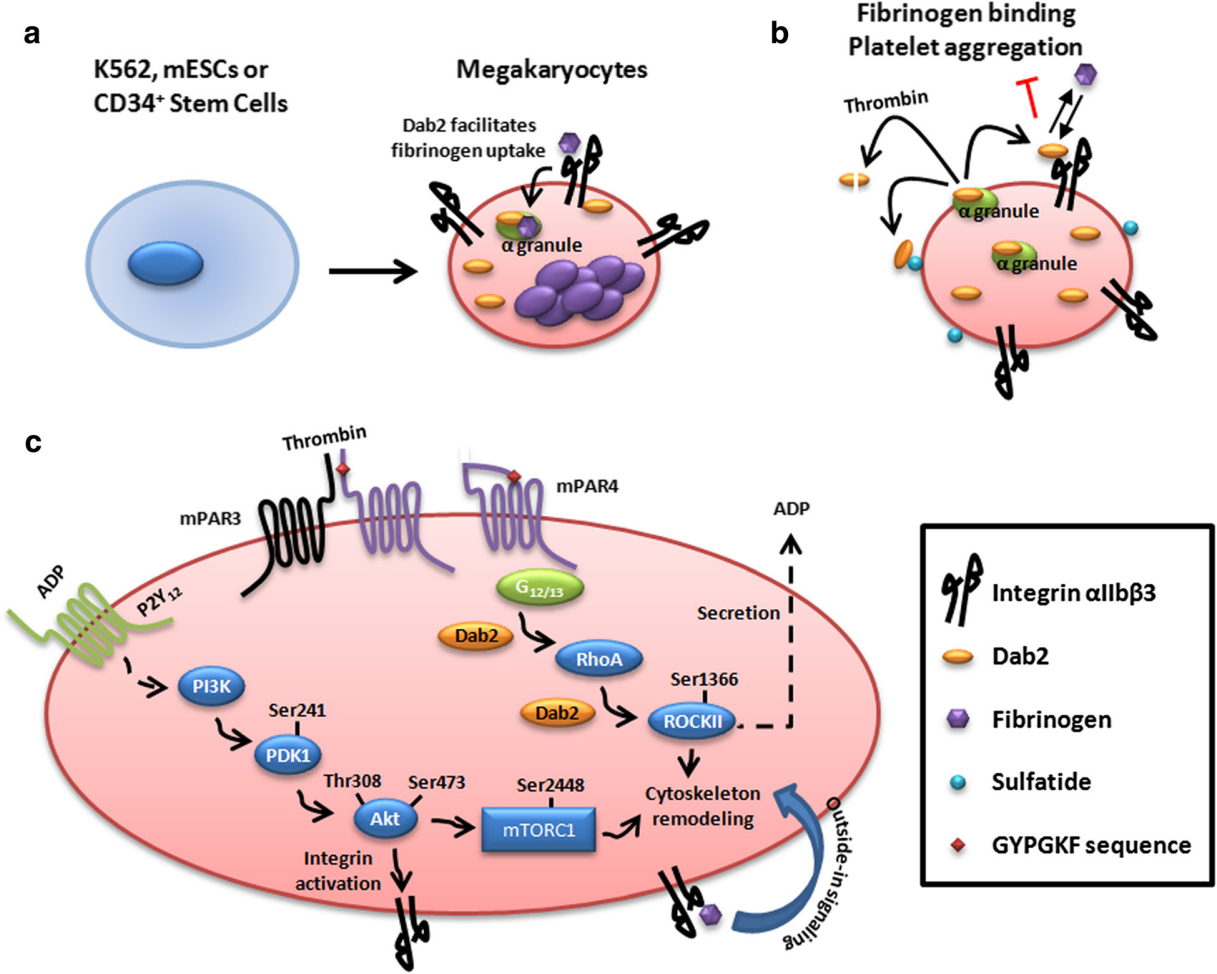
Schematic representation of Dab2 functions in megakaryocytic differentiation and platelet activation. **a** Dab2 is upregulated during megakaryocytic differentiation of K562 cells, mESC and CD34^+^ hematopoietic stem cells and is involved in fibrinogen uptake during megakaryocytic differentiation. **b** Dab2 is present in the cytoplasm and α-granules of platelets. By interacting with the intracellular and extracellular portions of αIIbβ3 integrin, Dab2 regulates platelet aggregation and fibrinogen binding induced by various platelet agonists with the exception of thrombin which is the protease of Dab2. The cleavage of Dab2 by thrombin is protected when Dab2 is associated with sulfatide. **c** Dab2 regulates the inside-out signaling of mouse platelets stimulated by low concentrations of thrombin

Dab2 has been shown to elicit multiple functions in megakaryocytic differentiation and platelet signaling (Table 1 and Fig. 2). The human K562 leukemic cells induced by TPA to form megakaryocyte-like cells have been used to define the role of Dab2 in integrin activation, cell adhesion, fibrinogen uptake, and megakaryocytic differentiation [31–34]. Dab2 is crucial in cell-cell adhesion of K562 cells and negatively regulates integrin αIIbβ3 activation and cell adhesion to fibrinogen. A mutual regulation between Dab2 and MAPK was also unveiled when the K562 cells are undergone megakaryocytic differentiation [31, 32]. Dab2 colocalizes with clathrin and mediates fibrinogen uptake in the primary megakaryocytes and megakaryocytic differentiating K562 cells [34]. Dab2-associated regulatory circuit controls mesoderm and megakaryocytic differentiation by regulating β-catenin and plakoglobin cellular distribution through the interaction between the PTB domain of Dab2 and the Asn-Pro-Asp-Tyr motif of plakoglobin [35]. Accordingly, down-regulation of Dab2 in murine ESCs disrupts cell-cell adhesion and affects embryoid body and colony formation leading to impaired mesodermal and megakaryocytic differentiation. Multiple roles of Dab2 in intracellular signaling, integrin activation and fibrinogen uptake of the megakaryocytic cells, and the fate determination of mesodermal and megakaryocytic differentiation were defined by these studies.

Dab2 is present in the cytoplasm and α-granules of human platelets and is released from the platelets in response to platelet activation [10]. Dab2 is shown in a number of studies playing a pivotal role in the activation of human platelets. Dab2 interacts with the cytoplasmic tail of the platelet integrin and regulates inside-out signaling [33]. On the other hand, Dab2 released from the α-granules is able to bind the extracellular region of αIIbβ3 integrin through the Dab2 cell-adhesion Arg-Gly-Asp (RGD) motif (amino acid residues 64–66) and the fibrinogen binding region of integrin αIIb. Such interactions compete for the integrin αIIb-fibrinogen interactions and inhibit platelet aggregation induced by soluble agonists except thrombin. Thrombin renders Dab2 inactive by cleavage of Dab2 at the amino acid residue 64 [10]. Notably, the action of thrombin on Dab2 cleavage is suppressed when Dab2 binds to the phospholipid sulfatide through four positively charged residues (Lys25, Lys49, Lys51, and Lys53) located at the PTB domain [47]. Hence, two pools of Dab2 is present at the outer surface of the platelet plasma membrane, one at the sulfatide-bound and the other at the integrin αIIbβ3-bound states. The balance between these two Dab2 states is involved in the regulation of clotting reaction, platelet aggregation and the interactions of platelet and fibrinogen in response to stimulation by platelet agonists [10, 47–49].

Megakaryocyte/platelet lineage-restricted Dab2 knockout (Dab2^-/-^) mice have been generated by using the Cre-loxP transgenic system driven by the platelet factor 4 promoter to elucidate Dab2 functions in vivo [9]. Dab2^-/-^ platelets, when stimulated by low concentrations of thrombin, are defective in platelet aggregation, clot retraction, and spreading on fibrinogen. The functional imperfection of Dab2^-/-^ platelets is related to the defective responses to thrombin-induced RhoA-ROCKII and Akt-mTOR activation, ADP release and integrin αIIbβ3 activation [9]. Although Dab2 elicits a restrictive function to the murine platelets stimulated by low concentrations of thrombin, defective Dab2 expression has profound effects on hemostasis and thrombosis in vivo. This is evidenced by the observations that bleeding time is prolonged and thrombus formation is impaired in the Dab2^-/-^ mice. These findings are consistent with the perception that protease-activated receptors (PAR) 3- and PAR4-deficient mice, despite eliciting a partial decrease in thrombin-induced platelet aggregation, are impaired in hemostasis and are protected against thrombosis [50, 51]. These studies support the notion that Dab2 is a key regulator in hemostasis and thrombosis.

### The consensuses, controversies and future prospects

The study linking Dab2 functions with megakaryocytic differentiation, platelet signaling and integrin activation was first reported 15 years ago [31]. The studies up-to-date support extensively that Dab2 is a regulator of megakaryocytic differentiation and platelet function. Nevertheless, distinctive functions of Dab2 in human and mouse megakaryocytes and platelets are noted. Knockdown of Dab2 results in an increase in αIIbβ3 activation and cell adhesion to fibrinogen in the K562 cells [33]. Dab2 is, however, required for murine platelet aggregation and integrin αIIbβ3 activation when the platelets are stimulated by low concentrations of thrombin [9]. Dab2 negatively regulates human platelet-fibrinogen interaction and platelet aggregation induced by soluble agonists except thrombin [10, 47–49]. The complexity in the abundance and species-specific expression of Dab2 isoforms and the diverse thrombin signaling in human and murine platelets [6, 9] likely contribute to the reported heterogeneity of Dab2 function. Alternatively, the discrepancy in these findings could be due to the different assay systems being used in these studies. In the studies of human platelets, recombinant Dab2 protein was the main tool for analyzing the effects of Dab2 function on platelet response by in vitro experiments [10, 47–49]. There are no Dab2 mutants or Dab2-deficient human platelets available for in vivo study. On the other hand, Dab2-deficient murine platelets have been generated for analysis of Dab2 function in vivo [9]. An animal model expressing human platelet Dab2 should help us to understand the distinctive Dab2 functions in human and murine platelets.

Dab2 is known as a phosphoprotein in a variety of cellular processes [12, 25, 33, 40–43]. Due to the lack of appropriate tools, the physiological functions of Dab2 phosphorylation in platelet signaling have not yet been elucidated. We have addressed these issues in our recent work and revealed that Dab2 is phosphorylated during agonist-stimulated human platelet activation (unpublished data). With the new tools such as CRISPR and TALEN [52–55] in the generation of genetically modified animals, the progress in gaining new insights into the signaling network involving Dab2 expression and phosphorylation in megakaryocyte, platelet biology and integrin signaling is expected to accelerate.

## Conclusions

Multiple functions of platelets in hemostasis, thrombosis, immunology, cancer progression, microorganism infections call for considerable attention on understanding how the activated platelets transmit intracellular signal to elicit their roles in different biological responses. Extensive evidence from the studies in the past decades demonstrates that Dab2 is a key regulator of platelet signaling, in particular, the endocytosis and the activation of thrombin-stimulated inside-out signaling of platelet integrin. With the complicated nature of the signaling network within megakaryocytes and platelets, the definitive roles of Dab2 in megakaryocytic differentiation, platelet activation and integrin signaling remain to be explored further.

## Abbreviations

ADP: Adenosine diphosphate
CSF-1: Colony-stimulating factor-1
Dab2: Disabled-2
ESCs: Embryonic stem cells
ITAM: Immunoreceptor tyrosine-based activation motif
PARs: Protease-activated receptors
PKC: Protein kinase C
PRD: Proline rich domain
PTB: Phosphotyrosine binding domain
TPA: 12-O-tetradecanoylphorbol-13-acetate
TXA_2_: Thromboxane A_2_
